# Prélèvement des kystes hydatiques par la méthode FTA Card pour la caractérisation moléculaire d’*Echinococcus granulosus* sensu lato en Algérie. Résultats préliminaires

**DOI:** 10.48327/mtsi.v3i3.2023.416

**Published:** 2023-09-04

**Authors:** Mohammed Chérif BENCHIKH EL FEGOUN, Gérald UMHANG, Franck BOUÉ, Karima KOHIL, Baaissa BABELHADJ, Saoussene RABHI, Rahma SLIMANI, Nazim MESSAOUDI, Abdelaziz AGUEZLANE, Abdelaziz ZOUIKRI

**Affiliations:** 1 Institut des sciences vétérinaires, Université des Frères Mentouri, 25000 Constantine, Algérie; 2 ANSES, Laboratoire Rage et faune sauvage de Nancy, Laboratoire national de référence pour *Echinococcus* spp., Unité Surveillance et éco-épidémiologie des animaux sauvages, Technopôle agricole et vétérinaire, CS 40009, 54220 Malzéville, France; 3 École normale supérieure d'Ouargla, Algérie

**Keywords:** Méthode de prélèvement, FTA Card, Typage moléculaire, *Echinococcus granulosus*, Kyste hydatique, Ruminants, Sétif, Jijel, Constantine, Oum El Bouaghi, Biskra, Ouargla, Algérie, Maghreb, Afrique du Nord, FTA Card sampling method, Molecular typing, *Echinococcus granulosus*, Hydatid cyst, Ruminants, Sétif, Jijel, Constantine, Oum El Bouaghi, Biskra, Ouargla, Algeria, Maghreb, Northern Africa

## Abstract

**Introduction et objectifs:**

L’échinococcose kystique est fortement endémique en Algérie et constitue un problème socio-économique majeur. Le typage des espèces du complexe *Echinococcus granulosus* sensu lato circulant chez les bovins nécessite de recourir à une méthode de prélèvement de kystes hydatiques adaptée aux conditions de terrain difficiles (chaleur et humidité élevées, temps de transport long). La méthode FTA Card constitue actuellement un moyen efficace de conservation des échantillons biologiques avant leur analyse moléculaire. Dans la présente étude, la méthode FTA Card a été utilisée dans le prélèvement des kystes hydatiques pour identifier les espèces d’*E. granulosus* sensu lato circulant chez les ruminants (hôtes intermédiaires) dans l'Est algérien.

**Matériel et méthodes:**

Une PCR a été réalisée, pour 41 échantillons de kystes hydatiques prélevés dans six abattoirs de l'Est algérien, ciblant le gène mitochondrial *cox1*. Les produits de PCR ont été visualisés par électrophorèse dans un gel d'agarose à 1%.

**Résultats et conclusion:**

Les résultats de l'analyse moléculaire de tous les échantillons de kystes hydatiques ont confirmé la présence d’*E. granulosus* sensu stricto chez les ovins, les bovins et les camelins. Le caractère ubiquitaire du génotype G1 a été démontré.

L'utilisation de l’échantillonnage sur FTA Card est une méthode efficace et simple pour obtenir un échantillon biologique afin de caractériser l'espèce d’*E. granulosus* sensu lato en Algérie. La bonne conservation de l'ADN dans cette matrice facilitera l'obtention de nouvelles données moléculaires à partir des régions difficiles. L'identification des espèces du complexe *E. granulosus* sensu lato impliquées dans le cycle biologique est un préalable essentiel à la mise en oeuvre des mesures de contrôle, car différentes espèces hôtes participent à leur cycle évolutif. La caractérisation des génotypes d’*E. granulosus* est essentielle pour définir une stratégie de lutte adaptée contre l’échinococcose kystique.

## Abstract (en)

**Introduction and objectives:**

Cystic echinococcosis is highly endemic in Algeria and constitutes a major socio-economic problem. Typing the species of the *Echinococcus granulosus* sensu lato complex circulating in cattle requires the use of a hydatid cyst sampling method adapted to difficult field conditions (high heat and humidity, long transport time). The FTA Card method currently constitutes an effective means of preserving biological samples before their molecular analysis. In the present study, the FTA Card method was used in the collection of hydatid cysts to identify the species of *E. granulosus* sensu lato circulating in ruminants (intermediate hosts) in eastern Algeria.

**Material and methods:**

A PCR was carried out for 41 samples of hydatid cysts taken from six slaughterhouses in eastern Algeria, targeting the *cox1* mitochondrial gene. PCR products were visualized by electrophoresis in a 1% agarose gel.

**Results and conclusion:**

The results of the molecular analysis of all hydatid cyst samples confirmed the presence of *E. granulosus* sensu stricto in sheep, cattle and camels. The ubiquitous nature of the G1 genotype has been demonstrated. The use of FTA Card sampling is an efficient and simple method to obtain a biological sample in order to characterize the species of *E. granulosus* sensu lato in Algeria. The good preservation of the DNA in this matrix will make it easier to obtain new molecular data from difficult regions. The identification of the species of the *E. granulosus* sensu lato complex involved in the biological cycle is an essential prerequisite for the implementation of control measures, since different host species participate in their evolutionary cycle. The characterization of *E. granulosus* genotypes is essential to define an appropriate control strategy against cystic echinococcosis.

## Introduction

L’échinococcose kystique est une zoonose hautement endémique dans de nombreuses régions du monde. Elle est causée par le stade larvaire du ténia, *Echinococcus granulosus*, un parasite des chiens domestiques et d'autres espèces de canidés sauvages. Le parasite se transmet selon un cycle synanthropique principalement entre les chiens, qui servent d'hôtes définitifs, et les moutons, qui servent d'hôtes intermédiaires [14, 18].

L’échinococcose kystique est un problème socio-économique et de santé publique majeur, en particulier dans les régions d’élevage ovin comme les pays d'Afrique du Nord [7, 8, 16]. Les pertes financières mondiales annuelles chez les humains et le bétail ont été estimées respectivement à 193 529 740 $ et 14 605 195 $ [6]. Cette zoonose majeure a également un impact indirect sur le développement socioéconomique des populations humaines dont le bétail constitue un important moyen de subsistance.

En Algérie, l’échinococcose kystique est endémique chez les humains avec une incidence chirurgicale annuelle allant de 1,78 à 2,26 pour 100 000 habitants [10, 15].

Pour caractériser les espèces du complexe *E. granulosus* sensu lato circulant chez le bétail et établir un programme de contrôle adapté, les prélèvements de kystes hydatiques en abattoir doivent être conservés dans de bonnes conditions avant leur analyse moléculaire en laboratoire. En Algérie, les conditions de terrain sont souvent difficiles pour la conservation des échantillons (chaleur et humidité élevées, temps de transport long). L'utilisation de la méthode FTA Card est actuellement un moyen efficace pour faciliter la conservation des échantillons biologiques avant leur analyse moléculaire.

La présente étude a porté sur l'utilisation de la méthode FTA Card dans le prélèvement des kystes hydatiques pour identifier les espèces d’*E. granulosus* sensu lato circulant chez les ruminants (hôtes intermédiaires) dans l'Est algérien.

## MATÉRIEL ET MÉTHODES

Les échantillons de kyste hydatique ont été prélevés en utilisant la méthode FTA Card. L’échantillon de kyste hydatique (membrane proligère) est appliqué sur du papier cartonné FTA et laissé à sécher; les cellules sont lysées au contact. L'ADN de haut poids moléculaire libéré est immobilisé dans la matrice et protégé contre les nucléases, l'oxydation et les effets nocifs des rayons UV.

**Figure 1:**
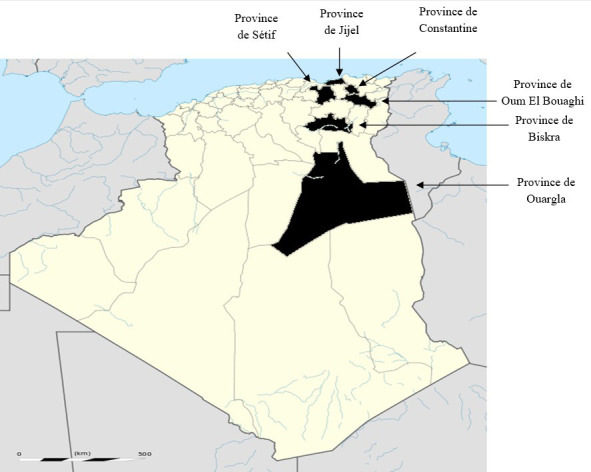
Situation géographique des régions d’étude Geographical location of the study regions

Les 41 échantillons prélevés en conditions de terrain dans différents abattoirs de l'Est algérien (Fig. 1) se répartissent en 24 kystes hydatiques de bovins, 13 kystes de moutons et 4 kystes de dromadaires, retrouvés dans le foie et les poumons. Le kit d’échantillon individuel contient une carte papier FTA, des gants et un formulaire pour enregistrer les informations relatives à l’échantillon. Après un minimum de 24 heures à température ambiante pour sécher les cartes FTA, les échantillons ont été envoyés au laboratoire de Parasitologie de l'Institut des sciences vétérinaires de Constantine (Algérie) où ils ont été conservés à température ambiante en atmosphère sèche. Les échantillons ont ensuite été envoyés au Laboratoire de référence pour *Echinococcus* spp. à Nancy, France (ANSES) pour l'analyse moléculaire.

Extraction d'ADN et amplification PCR

Un carré d'environ 1 cm^2^ a été découpé dans la partie colorée du papier FTA à l'aide d'un scalpel et d'une pince, différents pour chaque échantillon, afin d’éviter toute contamination. Chaque fragment a ensuite été placé dans un tube Eppendorf de 1,5 ml contenant 1 ml de tampon de lyse et 20 μL de protéinase K. Les tubes ont été vortexés et incubés à 55 °C pendant 1 heure 30 minutes. Le papier FTA a ensuite été retiré et l'ADN a été extrait directement du surnageant. Une PCR a été réalisée pour tous les échantillons, ciblant le gène mitochondrial *cox1* comme indiqué précédemment [19]. Les produits de PCR ont été visualisés par électrophorèse dans un gel d'agarose à 1% (p/v) coloré avec du SYBR Safe. Les amplicons ont été séquencés par une société privée (Beckmann Coulter Genomics) et les séquences nucléotidiques ont été analysées à l'aide du programme Vector NTI Software (Invitrogen, France) avant d’être comparées aux séquences obtenues dans GenBank à l'aide du programme BLASTn.

## Résultats et Discussion

L'amplification par PCR a été possible pour tous les échantillons de cartes FTA de l’étude expérimentale de kystes précédemment diagnostiqués réalisée en laboratoire. L'analyse de tous les échantillons a permis d'identifier l'espèce *E. granulosus* sensu stricto (Tableau I). L'utilisation de la technique d’échantillonnage sur FTA Card pour la détection par PCR du stade larvaire d’*Echinococcus* spp. a été validée avec succès sur des échantillons en laboratoire. L’évaluation en conditions de terrain en Algérie par prélèvement de kystes hydatiques parasitaires aux abattoirs a également donné des résultats satisfaisants.

**Tableau I:** Résultats de la PCR et identification des séquences après extraction selon la méthode FTA Card PCR results and sequence identification after extraction using FTA Card method

Abattoir	Espèce	Sexe	Âge	Localisation des kystes	Séquençage
BISKRA	Bovin	F	> 5 ans	Foie	*E. granulosus* sensu stricto (G1)
BISKRA	Bovin	M	15 mois	Foie	*E. granulosus* sensu stricto (G1)
BISKRA	Ovin	F	> 5 ans	Poumon	*E. granulosus* sensu stricto (G1)
BISKRA	Ovin	F	8 ans	Poumon	*E. granulosus* sensu stricto (G1)
BISKRA	Ovin	F	6 ans	Foie	*E. granulosus* sensu stricto (G1)
SÉTIF	Bovin	F	> 5 ans	Poumon	*E. granulosus* sensu stricto (G3)
SÉTIF	Bovin	F	> 5 ans	Poumon	*E. granulosus* sensu stricto (G3)
CONSTANTINE	Bovin	F	6 ans	Foie	*E. granulosus* sensu stricto (G1)
CONSTANTINE	Bovin	F	> 5 ans	Poumon	*E. granulosus* sensu stricto (G1)
SÉTIF	Bovin	F	> 5 ans	Foie + Poumon	*E. granulosus* sensu stricto (G1)
BISKRA	Ovin	F	> 5 ans	Poumon	*E. granulosus* sensu stricto (G1)
BISKRA	Ovin	M	18 mois	Foie	*E. granulosus* sensu stricto (G1)
CONSTANTINE	Bovin	F	> 5 ans	Poumon	*E. granulosus* sensu stricto (G1)
BISKRA	Ovin	F	> 5 ans	Foie	*E. granulosus* sensu stricto (G1)
CONSTANTINE	Bovin	F	8 ans	Poumon	*E. granulosus* sensu stricto (G1)
SÉTIF	Ovin	F	> 2 ans	Foie	*E. granulosus* sensu stricto (G1)
SÉTIF	Bovin	F	> 5 ans	Foie	*E. granulosus* sensu stricto (G3)
JIJEL	Bovin	M	4 ans	Foie	*E. granulosus* sensu stricto (G1)
JIJEL	Bovin	F	10 ans	Foie	*E. granulosus* sensu stricto (G1)
JIJEL	Ovin	M	2 ans	Foie	*E. granulosus* sensu stricto (G1)
JIJEL	Bovin	F	10 ans	Foie + Poumon	*E. granulosus* sensu stricto (G1)
JIJEL	Bovin	M	2 ans	Foie	*E. granulosus* sensu stricto (G1)
SÉTIF	Bovin	F	> 5 ans	Poumon	*E. granulosus* sensu stricto (G1)
BISKRA	Bovin	M	15 mois	Poumon	*E. granulosus* sensu stricto (G1)
BISKRA	Bovin	F	> 5 ans	Poumon	*E. granulosus* sensu stricto (G1)
BISKRA	Bovin	M	18 mois	Poumon	*E. granulosus* sensu stricto (G1)
JIJEL	Bovin	M	24 mois	Poumon	*E. granulosus* sensu stricto (G1)
JIJEL	Bovin	F	8 ans	Poumon	*E. granulosus* sensu stricto (G3)
OUM EL BOUAGHI	Bovin	F	> 5 ans	Poumon	*E. granulosus* sensu stricto (G1)
OUM EL BOUAGHI	Bovin	F	> 5 ans	Poumon	*E. granulosus* sensu stricto (G1)
OUM EL BOUAGHI	Ovin	/	> 2 ans	Foie	*E. granulosus* sensu stricto (G1)
SÉTIF	Bovin	F	> 5 ans	Poumon	*E. granulosus* sensu stricto (G1)
SÉTIF	Ovin	F	> 2 ans	Foie	*E. granulosus* sensu stricto (G1)
JIJEL	Bovin	M	2 ans	Rein	*E. granulosus* sensu stricto (G1)
OUARGLA	Camelin	F	5 ans	Poumon	*E. granulosus* sensu stricto (G1)
OUARGLA	Camelin	F	6 ans	Poumon	*E. granulosus* sensu stricto (G1)
OUARGLA	Camelin	F	4 ans	Poumon	*E. granulosus* sensu stricto (G1)
OUARGLA	Camelin	F	7 ans	Poumon	*E. granulosus* sensu stricto (G1)
OUARGLA	Ovin	F	5 ans	Foie	*E. granulosus* sensu stricto (G1)
OUARGLA	Ovin	F	4 ans	Poumon	*E. granulosus* sensu stricto (G1)
OUARGLA	Ovin	F	3 ans	Foie	*E. granulosus* sensu stricto (G1)

En Algérie, la méthode FTA Card est fortement indiquée dans des conditions où la collecte et la conservation des échantillons biologiques sont particulièrement difficiles. Cette méthodologie d’échantillonnage s'est révélée efficace pour un panel d'animaux (hôtes intermédiaires).

Dans la présente étude, les résultats d'analyses moléculaires d’échantillons de kystes hydatiques prélevés dans six abattoirs de l'Est algérien où la méthode FTA Card a été utilisée, ont confirmé la présence d’*E. granulosus* sensu stricto (G1) chez les ovins et les bovins [2,3,4,13], mais aussi chez les dromadaires [11]. Dans le sud de l'Algérie, *Echinococcus granulosus canadensis* (G6) a également été identifié chez les dromadaires dans une précédente étude [11].

La présence d’*E. granulosus* s.s. chez les ruminants (hôtes intermédiaires) a également été signalée dans des enquêtes menées dans des abattoirs au Maroc, où la méthode FTA Card a été utilisée avec succès [1, 9]. Le caractère ubiquitaire d’*E. granulosus* sensu stricto (génotype G1) a été rapporté par Tashani *et al.* en Libye [17], Lahmar *et al.* en Tunisie [12], Azlaf *et al.* au Maroc [1], ainsi que Bardonnet *et al.* [2], Benchikh El Fegoun [4] et Kohil *et al.* en Algérie [11]. En effet, le génotype G1 circule chez les ovins, les bovins, les caprins et les camelins. La présence du génotype G1 chez plusieurs espèces animales suggère l'existence d'un polymorphisme génotypique.

## Conclusion

L'utilisation de l’échantillonnage sur FTA Card est une méthode efficace et simple pour obtenir un échantillon biologique afin de caractériser l'espèce *E*. *granulosus* sensu lato en Algérie. La bonne conservation de l'ADN dans cette matrice facilitera l'obtention de nouvelles données moléculaires à partir de régions difficiles et/ou isolées. La technique FTA Card s'est avérée efficace dans l'identification des espèces d*’E. granulosus* sensu lato au Maroc, au Mali et en Mauritanie [5]. L'identification d’*E. granulosus* sensu lato impliqué dans le cycle biologique est un préalable essentiel à la mise en place de mesures de contrôle, car différentes espèces hôtes participent à leur cycle évolutif. La caractérisation des génotypes d’*E. granulosus* est essentielle pour définir une stratégie de lutte adaptée contre l’échinococcose kystique. De plus, l'hétérogénéité du complexe *E. granulosus* sensu lato peut influencer les modèles de cycle biologique, la spécificité de l'hôte, la période prépatente, la dynamique de transmission, la contamination humaine et l'antigénicité (Ag G1 ≠ Ag G6).

## CONTRIBUTION DES AUTEURS

- Prélèvements des échantillons de kystes hydatiques dans les différents abattoirs de l'Est algérien : B. BABELHADJ, S. RABHI, R. SLIMANI, N. MESSAOUDI, A. AGUEZLANE, A. ZOUIKRI

- Utilisation de la méthode FTA card sur les échantillons de kystes hydatiques prélevés : M. C. BENCHIKH EL FEGOUN et K. KOHIL

- Analyse moléculaire des échantillons pour l'identification des espèces d’*Echinococcus granulosus* sensu lato : G. UMHANG, F. BOUÉ

- Rédaction de l'article, tableau et figure : M. C. BENCHIKH EL FEGOUN

## LIENS D'INTÉRÊTS

Les auteurs déclarent n'avoir aucun conflit d'intérêts.
